# Comparative Effect of 22 Dietary Sources of Fiber on Gut Microbiota of Healthy Humans *in vitro*

**DOI:** 10.3389/fnut.2021.700571

**Published:** 2021-07-02

**Authors:** Marta Calatayud, Pieter Van den Abbeele, Jonas Ghyselinck, Massimo Marzorati, Eric Rohs, Anne Birkett

**Affiliations:** ^1^ProDigest, Ghent, Belgium; ^2^Faculty of Bioscience Engineering, Center for Microbial Ecology and Technology (CMET), Ghent University, Ghent, Belgium; ^3^Kellogg Company, Battle Creek, MI, United States

**Keywords:** dietary fiber, gut microbiota, fermentation, *in vitro*, short-chain fatty acids, cereals, seeds, pulses

## Abstract

Human gut microbiota has a fundamental role in human health, and diet is one of the most relevant factors modulating the gut microbial ecosystem. Fiber, fat, proteins, and micronutrients can shape microbial activity and structure. Much information is available on the role of defined prebiotic fibers on gut microbiota, but less known are the effects of intact dietary fiber sources on healthy gut ecosystems. This research investigated *in vitro* the short-term effect of 22 commercially available food sources of dietary fiber on gut microbiota activity [pH, gas, short-chain fatty acids (SCFA), branched fatty acids (BCFA), lactate] and specific composition of Firmicutes, Bacteroidetes, bifidobacteria, and lactobacilli populations. More than 80% (19 of 22) of the products were highly fermentable and induced SCFAs production, with specific product differences. In general, all the whole grain cereals had a similar effect on gut microbiota modulation, inducing acetate and butyrate production and increasing bifidobacteria levels. Incorporating and comparing a large variety of products, including “non-conventional” fiber sources, like konjac, bamboo fiber, or seeds fiber, about which there is little information, contributes to our knowledge on the modulatory activity of diverse food fiber sources on human gut microbiota, and therefore potential health promotion through dietary fiber diversification.

## Introduction

Dietary fiber is composed of carbohydrate polymers with three or more monomeric units, which are not digested or absorbed in the human small intestine (1–3). It is a heterogeneous group, mostly originated from plant foods, and varying by composition, architecture, and functionality. Dietary fibers include resistant oligosaccharides, non-starch polysaccharides, resistant starches, and some non-carbohydrate substances such as lignin.

Dietary fiber intake has been associated with multiple health benefits, including reduced risk for heart disease, stroke, hypertension, specific gastrointestinal disorders, obesity, type 2 diabetes, and some cancers (4). Codex Alimentarius includes decreased intestinal transit time, increased stool bulk, fermentation by colonic microbiota, reduction of blood total and/or LDL cholesterol levels, and reduced postprandial blood glucose/insulin levels as health benefits of dietary fiber (5). Currently, dietary fibers with EFSA-approved health claims include (a) plant sources of fiber (e.g., wheat bran, barley grain, rye, oat grain, oat beta glucan) and (b) specific fiber types (e.g., beta-glucan, cellulose, arabinoxylan, glucomannan, pectin, guar gum, inulin, and resistant starch) (6).

Due to health benefits associated with dietary fiber intake, multiple organizations such as the UK Scientific Advisory Committee on Nutrition (7), the United States Institute of Medicine (IOM) (8), and the European Food Safety Agency (EFSA) (9) have developed recommendations for dietary fiber consumption (for adults—SACN 30 g/day, IOM 14 g/1,000 kcal, EFSA 25 g/day) as part of a public health strategy to promote health and reduce risk of chronic disease (4). Since 2005, fiber has been considered a “nutrient of concern” by the Dietary Guidelines for Americans because it is under-consumed by at least nine of ten adults; similarly, it is under-consumed in most developed countries, with most adults not meeting their recommended fiber intake (4, 10).

Reasons for the fiber intake shortfall are multifactorial, including consumer misperception on dietary sources of fiber and adequate intake amounts, changing dietary habits toward Western, high-protein, gluten-free, wheat-free, or grain-free diets, or gastrointestinal discomfort associated with gas production after increasing fiber intake (4).

Some of the health benefits of dietary fiber can be linked to the gut microbiota. Dietary fibers undergo bacterial fermentation upon arriving in the colon and thus impact the composition and functionality of bacterial communities, including the production of fermentative metabolites with different effects on the host (11). Gut dysbiosis, described as the loss of keystone taxa, loss of diversity, alterations in metabolic capacity, or pathogen(s) overgrowth (12) have been connected to different metabolic and autoimmune pathological conditions (13, 14). Diet is one of the main modulators of gut microbiota structure and function; dietary fibers can induce specific growth and/or activity of gut bacterial populations (15), a promising strategy to maintain gut microbiota homeostasis and prevent non-communicable diseases.

Whole grains, vegetables, legumes, and fruit intrinsically contain fiber and provide most of the fiber to the diet. The major cereals consumed worldwide are wheat, milled rice, and maize, followed in production by oat, rye, barley, triticale, millet, and sorghum. Cereals are the leading staple food and considering that whole grain cereals contain an average amount of 7% of TDF could provide the recommended daily intake of fiber per day if consumed in the appropriate form. Other fiber source groups like pulses have declined in consumption per capita due to changes in dietary changes, consumer preferences, and domestic production failure in developing countries (16). Pulses are a subgroup of legume crops harvested for dried grains and containing high levels of total dietary fiber (10–34 g/100 g DW) and protein (21–25% w/w) (17–19).

Additionally, isolated and synthesized fibers can be added to foods or consumed as food supplements, to augment intake, with beneficial physiological effects. Prebiotics are a specific type of fiber, defined as a substrate that is selectively utilized by host microorganisms conferring a health benefit (20). Some foods contain prebiotic fibers; however, most of the research in the field of microbiota modulation by prebiotics has been done with purified fibers, functional products, or mixtures, while less information is available on the effect of commonly available food sources of fibers on gut microbiota modulation. Mechanistic assessment of shifts induced on gut microbiota by fiber intake is hampered due to intrinsic interindividual variability of gut microbiota and confounding dietary factors such as variations in fat, protein, and sugar intake. In that sense, *in vitro* models mimicking colonic ecosystems provide a useful means to screen the effect of specific dietary components on gut microbiota under controlled conditions.

We selected a wide variety of food sources of fiber (22 products) and tested in parallel the effect on gut microbiota activity [pH, gas, SCFA, lactate] and specific composition of Firmicutes, Bacteroidetes, bifidobacteria, and lactobacilli *in vitro*, including the interindividual variability of three healthy donors. Incorporating and comparing a large variety of products, including “non-conventional” fiber sources, about which there is little information, contributes to our knowledge on the modulatory activity of diverse food fiber sources on human gut microbiota.

## Materials and Methods

### Test Products

Description of the 22 fiber sources used in gastrointestinal digestion and colonic fermentation is shown in Table 1. Products were obtained from different suppliers in 2019 and grouped into five categories according to the fiber origin. As most of the selected fiber sources are cooked prior to eating, the products were cooked using standard techniques to mimic typical food processing (see Table 1).

**Table 1:** Description of products used in this research.

**Fiber group**	**Code**	**Plant source**	**Supplier**	**Supplier code**	**Plant source details**	**Processing conditions**
Whole grain cereals (WG)	WG1	Whole grain millet	Woodland Foods	G07	Whole grain	15 lb batch of grains cooked with water and steam in a Kellogg-designed pressurized rotary cooker for 50 min at 121°C. Then dried on a Kellogg-designed bed dryer with forced air at 116°C for 90 min.
	WG2	Whole grain oat	Richardson Milling	RM-G001	Whole grain	
	WG3	Medium grain whole brown rice	Gulf Rice	BRMG2-4	Whole grain	
	WG4	Non waxy whole grain soft white wheat	Adams	G1290	Whole grain	
	WG5	Whole grain barley (tamalpais/hulless)	Adams	G1444	Whole grain	
	WG6	Whole grain corn	Grain Millers	F75WG	Whole grain	
	WG7	Waxy whole grain soft white wheat	Adams	G1448	Whole grain	
	WG8	Waxy hulless barley	Adams	4000	Whole grain	
Cereals (non-whole grain) (C)	C1	Oat beta glucan	Lantmännen	PromOat	Oat Bran, Nordic Oats	Heated at 121°C using a Lincoln Impinger oven for 10 min.
	C2	Rice fiber	Rettenmaier	Vitacel RF310	Hull	
Seeds (S)	S1	Whole brown flaxseed	Hesco /HFI	CS362	Whole seed	Dried on a Kellogg-designed bed dryer with forced air at 116°C for 30 min.
	S2	Hemp seed	Woodland Foods	N73	Shelled hemp seed	
	S3	Psyllium fiber	Perrigo	125PH-1-1	Seed husk	Heated at 121°C using a Lincoln Impinger oven for 10 min.
Pulses (P)	P1	Whole brown lentils	Woodland Foods	B27	Whole lentil	15 lb batch of grains cooked with water and steam in a Kellogg-designed pressurized rotary cooker for 50 min at 121*C. Then dried on a Kellogg-designed bed dryer with forced air at 116*C for 90 min.
	P2	Soy fiber	Dupont	Fibrim 2000	Cell wall material of soybean cotyledon	Heated at 121°C using a Lincoln Impinger oven for 10 min.
	P3	Pea fiber	Roquette	I 50 M	Hull of yellow pea	
Other fibers (F)	F1	Kiwi fiber	AIDP	Livaux 5228-00-F	Gold Kiwi Fruit. Peeled, seeds removed. Freeze-dried flesh	Not processed at all—heat-sensitive
	F2	Inulin	Beneo	Orafti GR	Chicory roo	Heated at 121°C using a Lincoln Impinger oven for 10 min.
	F3	Bamboo fiber	Natural Fiber Solutions	Unicell BF200	Whole plant crushed, fiber-extracted	
	F4	Konjac flour	Dupont	Nutricol ME 8731	Root of tuber	
	F5	Apple fiber	Natural Fiber Solutions	AP200	Crushed apples, fiber-extracted.	
	F6	Algal beta glucan isolate	Noblegen	Eunite	*Euglena gracilis*, with ≥ 95% paramylon	

### Nutritional Content and Total Fiber Quantification

For each product, nutritional values reported in the product datasheets were recorded and summarized in Supplementary Table 1. Dietary fiber content reported by the provider was also confirmed using the McCleary method (21) in an ISO 17025 accredited laboratory (Eurofins Food Integrity Innovation, Madison, Wisconsin, United States). The difference between fiber content obtained by the McCleary method and dietary fiber content reported in the product datasheet was calculated as Δ(F_McC_−F_p_).

### Simulation of the Upper Gastrointestinal Digestion

Fifteen of the 22 test products (Supplementary Table 2) were subjected to a predigestion step consisting of a full passage through the oral, gastric, and small intestinal phase, including a dialysis step, before entering the simulating colonic environment. Products selected for predigestion were those having a sum of protein/fat/other carbohydrates exceeding 15% (w/w).

The *in vitro* gastrointestinal digestion was based on the consensus digestion protocol, from European-framework COST Action Infogest (22), with some modifications. The oral phase was implemented as proposed by Mackie et al. (23). Briefly, the food matrix was ground until obtaining particles <2 mm, and 5 g of product was subsequently mixed with simulated salivary fluid (1:4 w/v), including amylase (Sigma) and CaCl_2_ (AnalaR Normapur) at 75 U ml^−1^ and 0.75 mM in the final mixture, respectively (24). The suspension was vortexed for 30 s and incubated for 2 min, 90 rpm at 37°C. Then, simulated gastric fluid was added to the samples (1:1 v/v), pH was adjusted to pH 2 with HCl 1M (Carl Roth), and porcine pepsin (Chem Lab) and lecithin (Carl Roth) were added to a final concentration of 2,000 U ml^−1^ and 1.17 mM in the final digestion mixture, respectively. During gastric incubation (2 h), pH was measured every 15 min and adjusted to pH 2 when required.

After the gastric incubation, small intestinal fluid was added to the simulated gastric chyme (1:1 v/v), including pancreatin from porcine pancreas (100 U ml^−1^ of trypsin in the final mixture) (Sigma) and bile salts (10 mM, Oxoid), pH adjusted to 5.5 and incubated at 90 rpm for 30 min, simulating the duodenal digestion. Then, a dialysis approach was applied to simulate the small intestinal absorption. Briefly, 3.5-kDa dialysis membrane (ZelluTrans/Roth dialysis membranes, Carl Roth) was filled with small intestinal digest adjusted to pH 7 and submerged in dialysis fluid (3.75 g/L NaHCO3, pH 7) at 37°C on a shaker for 3 h, with the adjustment of pH of the intestinal content and replacement of the dialysis fluid every 45 min. At the end of the dialysis, the remaining intestinal solution was weighed and used for subsequent colonic incubations. Digestions were performed in single reactors for each product.

One of the products (F1, kiwi fiber), with a sugar content above 10 g/100 g, was dialyzed through a 0.5-kDa membrane without gastrointestinal predigestion.

### Experimental Design of Short-Term Colonic Incubations

**Figure 1:**
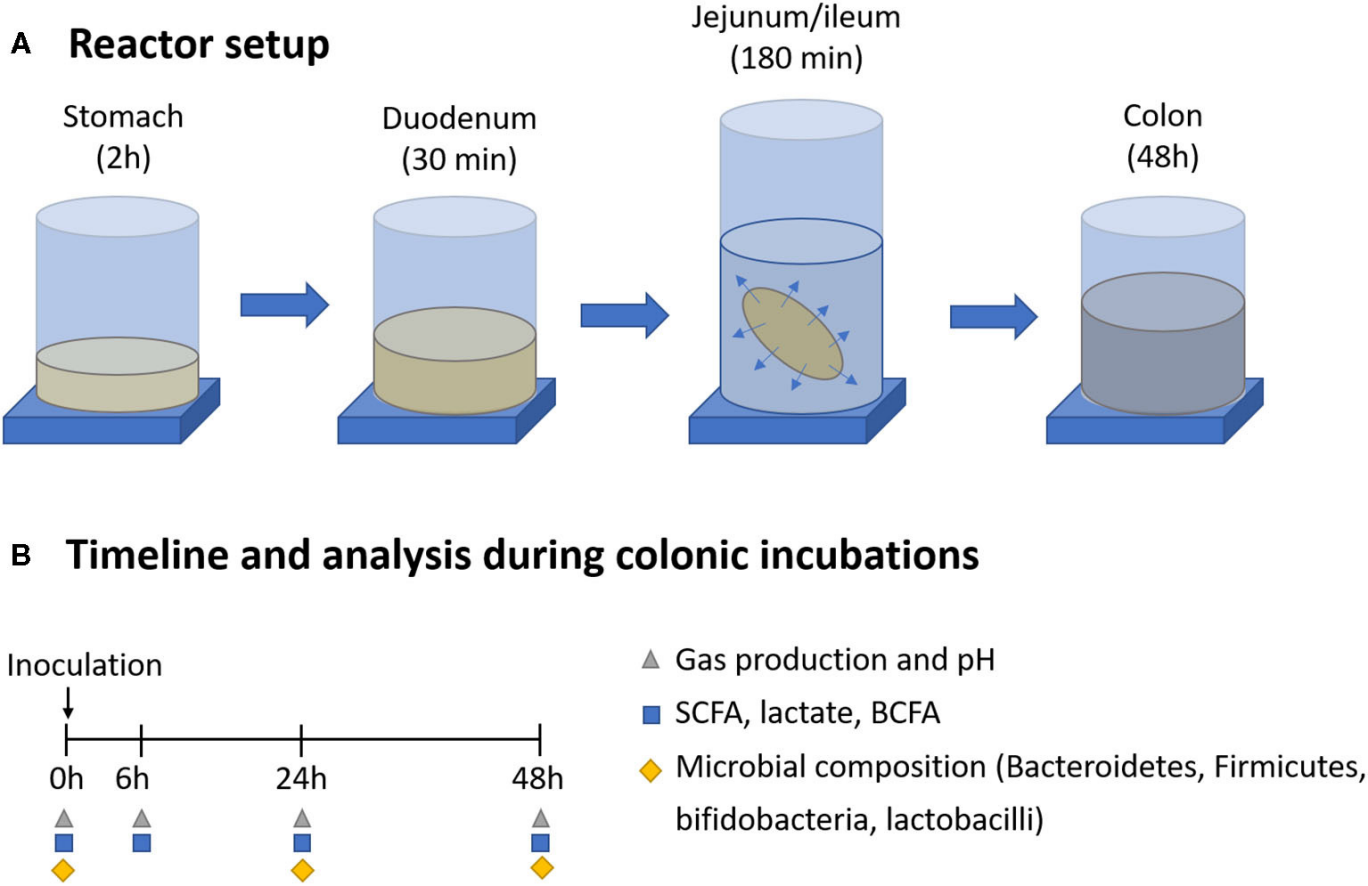
Overview of the gastrointestinal digestion and colonic incubation **(A)**, including sampling points and downstream analysis **(B)**.

The short-term screening assay consisted of a simulated colonic incubation of a single dose of the selected fiber source under conditions representative for the proximal colon region of an adult human, using representative bacterial inocula from three healthy donors (Figure 1).

Short-term fecal batch incubations were performed as previously described in Van den Abbeele et al. (25). Briefly, fresh fecal materials from three healthy human donors (29–35 y, 1 male, 2 females) were collected and separately homogenized in anaerobic phosphate buffer (K_2_HPO_4_ 8.8 g/L; KH_2_PO_4_ 6.8 g/L; sodium thioglycolate 0.1 g/L; sodium dithionite 0.015 g/L) (1:5 w/v) using a stomacher bagmixer for 10 min (BagMixer 400, Interscience, Louvain-LaNeuve, Belgium). Donors were selected based on the following inclusion criteria: age between 25 and 45 years, healthy, no antibiotic intake for at least 6 months before sampling, and consuming a self-selected standard Western diet. Samples were obtained followed by ethical approval of the University Hospital Ghent, reference number B670201836585. Big particles were removed by centrifugation (2 min, 500 g), and the fecal slurries (10% v/v) were used as inoculum for the colonic incubations. The fecal slurry obtained from each donor was added to prefilled penicillin bottles containing the dialyzed and non-dialyzed test ingredient products (5 g/L) (concentration assuming no product absorption during dialysis) and anaerobic sugar-depleted nutritional medium containing 3.5 g/L K_2_HPO_4_, 10.9 g/L KH_2_PO_4_, 2 g/L NaHCO_3_ (Chem-lab NV, Zedelgem, Belgium), 2 g/L yeast extract, 2 g/L peptone (Oxoid, Aalst, Belgium), 1 g/L mucin (Carl Roth, Karlsruhe, Germany), 0.5 g/L L-cysteine, and 2 ml/L Tween80 (Sigma-Aldrich, Bornem, Belgium). A control containing only the sugar-depleted nutritional medium (without product) was included per donor, allowing to assess the background activity of the bacterial community. Incubations were performed in anaerobic conditions for 48 h at 37°C, 90 rpm, and samples were collected at different time points (0, 6, 24, and 48 h) for the analysis of markers of overall microbial activity and microbial composition through qPCR of specific members of the gut microbiota (Firmicutes, Bacteroidetes, bifidobacteria, and lactobacilli) (0, 24, and 48 h).

### Microbial Community Analysis by qPCR

Samples collected after 24 and 48 h of incubation were evaluated for the total amount of Firmicutes, Bacteroidetes, bifidobacteria, and lactobacilli by qPCR. The DNA was extracted from a pellet of bacterial cells originated from a 1 ml sample after centrifugation for 5 min at 7,700 g. A Fastprep-24 device (MP BioMedicals, Illkirch, France) was used for homogenization (two cycles of 40 s at 4 m/s). Subsequently, the qPCR assays were performed using a StepOnePlus Real-Time PCR system (Applied Biosystems, Foster City, CA), using the primers and conditions described in Supplementary Tables 3, 4, adapted from Rinttilä et al. (26), Guo et al. (27), and Furet et al. (28). Each sample was analyzed in technical triplicate, and outliers (more than 1 CT difference) were omitted. The samples were checked for correcting melt curve peaks. The standard curves for all of the different runs had efficiencies between 90 and 105%. Results are reported as log (16S rRNA gene copies/ml).

### Microbial Metabolic Activity Analysis

The pH (Senseline F410; ProSense, Oosterhout, The Netherlands), gas (hand-held pressure indicator CPH6200; Wika, Echt, The Netherlands), lactate, and short-chain fatty acid (SCFA) measurements were taken at 0, 6, 24, and 48 h after starting the colonic incubation. Acetate, propionate, butyrate, and branched SCFAs (isobutyrate, isovalerate, and isocaproate) were measured as described by De Weirdt et al. (29). Lactate quantification was performed using a commercially available enzymatic assay kit (R-Biopharm, Darmstadt, Germany) according to the manufacturer's instructions. Data obtained from each donor, product, and time were measured in single, considering each donor as a biological replicate.

### Statistical Analysis

Each donor (n = 3)/product (n = 22) combination was tested in single reactors, and different donors were considered biological triplicates. To calculate statistically significant differences between groups of products by category, data from each category (control, whole grain cereals, non-whole grain cereals, seeds, pulses, other fibers) were pooled.

Delta values for pH, gas, SCFA, BCFA, and copies/ml obtained from qPCR were calculated by subtracting values obtained at the end of the experiment (48 h) minus values at the starting of the experiment (0 h) (Δ48 h). Relative values to control condition were calculated by the values obtained in each treatment minus the values in the control condition (rΔ48 h). Firmicutes/Bacteroidetes ratio was calculated using Δ48-h data.

All statistical analyses were performed in GraphPad Prism 9.0.0 (GraphPad Software, San Diego, CA) in relative Δ48 h. Significance level was set at 0.05. Normality of the dataset was tested with the Kolmogorov–Smirnov test. In the case of normality, the mean values of two different groups were compared with an independent samples t-test. Significant differences in microbial metabolites between different groups of fibers were tested with one-way ANOVA in the case of normality. Homogeneity of variances was tested with the modified Levene's test. Depending on the outcome of the Levene's test, Bonferroni or Dunnett's T3 was used as *post-hoc* test to determine p-values. In case of non-normal distributions, differences were tested with the nonparametric Mann–Whitney U-test.

Principal component analysis (PCA) plots were obtained by centering and scaling dimensions in ClustVis (http://biit.cs.ut.ee/clustvist/) (30), in order to reduce the impact of large units. Principal components were calculated using the singular value decomposition (SVD) method with imputation in pcaMethods (31). R package, which performs imputation and SVD iteratively until estimates of missing values converge. For the data processing, the unit variance scaling method was performed using the pcaMethods R package, which divides the values by SD so that each row has variance equal to one. Variables were grouped per treatment and sampling time, including data from three donors as biological triplicates.

## Results

### Descriptive Characterization of Nutritional Composition From Fiber-Rich Commercially Available Products

The whole grain cereal group showed a homogenous nutritional composition, with values of protein (10.9 ± 2.4%), fat (3.2 ± 1.6%), dietary fiber (12.5 ± 5.7%), sugars (0.6 ± 0.3%), and other carbohydrates (60.9 ± 8.1%) in the same range for all the different products (Supplementary Table 1).

The other fiber groups were compositionally heterogeneous. Within the non-whole grain cereals, C1 (oat beta glucan) had a 33% lower fiber content (65.0%) than C2 (rice fiber) (93.1%). Within the pulse group, P2 (soy fiber) and P3 (pea fiber) had a similar nutritional content in terms of protein (8–12%), fat (1–1.1%), dietary fiber (75–82%), sugars (n.d.−0.1%), and other carbohydrates (n.d.−3%). P1 (whole brown lentils), however, had a higher content of protein (24.6%) and other carbohydrates (50.6%). In the seeds group, the highest fat content (45.5 ± 4.7%) was found in S1 (whole brown flaxseed) and S2 (hemp seed). S3 (psyllium) showed a different nutritional profile compared to other seeds, with lower fat (0.8%) and protein (2.7%), and higher fiber content (97.1%).

When comparing dietary fiber levels described in the product datasheets with total fiber content quantified by the McCleary method, results were typically in agreement, with variation <20% (Supplementary Table 1), except for C1 (oat beta glucan). C1 had a reported fiber content of 65%, while the analyzed total fiber value was 33%.

### Several Fiber Sources (19/22) Shift Gut Microbiota Activity and Structure

**Figure 2:**
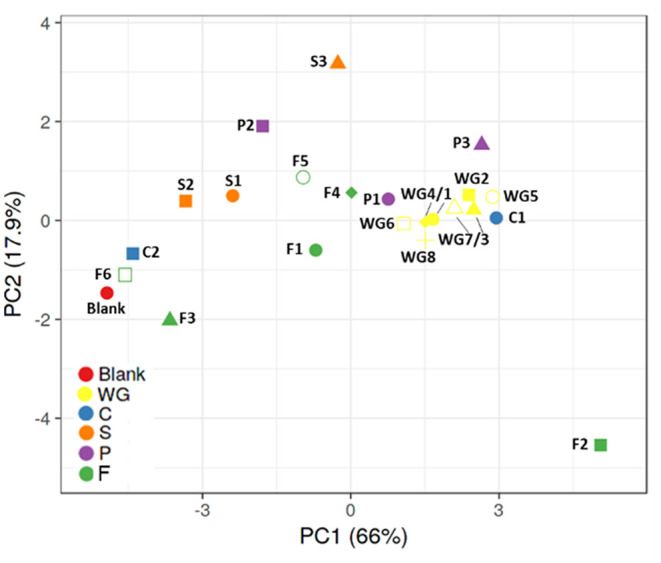
Principal component analysis (PCA) plots representing metabolic (pH, gas, SCFA, BCFA, lactate) and qPCR (bifidobacteria, lactobacilli, Bacteroidetes, Firmicutes) data obtained for batch assays with fecal samples of three healthy donors exposed to control and 22 fiber products. BCFA, branched-chain fatty acid; SCFA, short-chain fatty acid. WG1, whole grain millet; WG2, whole grain oat; WG3, medium grain whole brown rice; WG4, whole grain soft white wheat; WG5, whole grain barley; WG6, whole grain corn; WG7, waxy whole grain soft white wheat; WG8, waxy hulless barley; C1, oat beta glucan; C2, rice fiber; S1, whole brown flaxseed; S2, hemp/hemp hearts; S3, psyllium fiber; P1, whole brown lentils; P2, soy fiber; P3, pea fiber; F1, kiwi fiber; F2, inulin; F3, bamboo fiber; F4, konjac flour; F5, apple fiber; F6, algal beta glucan isolate.

Considering the PCA plot including metabolic (gas, pH, SCFA, BCFA, lactate) and qPCR data (Firmicutes, Bacteroidetes, bifidobacteria, lactobacilli), control condition without fermentable substrates is distant from most of the products, except C2 (rice fiber), F3 (bamboo fiber), and F6 (algal beta glucan isolate) (Figure 2). Whole grain cereals consistently grouped together and distant from the control condition, suggesting a potential similar fermentation capacity of healthy microbiota toward cereal substrates. Within the other fiber groups, there was higher intragroup variability, potentially due to a higher heterogenicity in product composition. Concretely, C1 (oat β-glucan) and P1 (whole brown lentils) induced similar shifts to those observed for whole grains, while C2 (rice fiber) was poorly fermented and clustered close to the control and F6 (algal beta glucan isolate). P3 (pea fiber) strongly stimulated the production of acetate and propionate and induced the inhibition of BCFA, with the highest effect on Bacteroidetes.

S1 (whole brown flaxseed) and S2 (hemp seed) are both high-fat-containing products, with fat% >10 and >40%, respectively, and had an intermediate pattern between the control and whole grains. S3 (psyllium fiber) had a different effect, not comparable to other groups or individual fibers, mainly due to a strong stimulation of propionate production, likely due to fermentation by increased levels of Bacteroidetes.

Within the entire group of test products, there was a high intragroup variability. F2 (inulin) is distant from other groups and control condition, indicating it is strongly fermentable, inducing significant lactate accumulation, BCFA inhibition, propionate production, and stimulatory potential on bifidobacteria and lactobacilli. F1 (kiwi fiber), F4 (konjac flour), and F5 (apple fiber) clustered more centrally within these extremes.

### Modulatory Effect of Dietary Fibers on Specific Members of the Human Gut Microbiota

**Figure 3:**
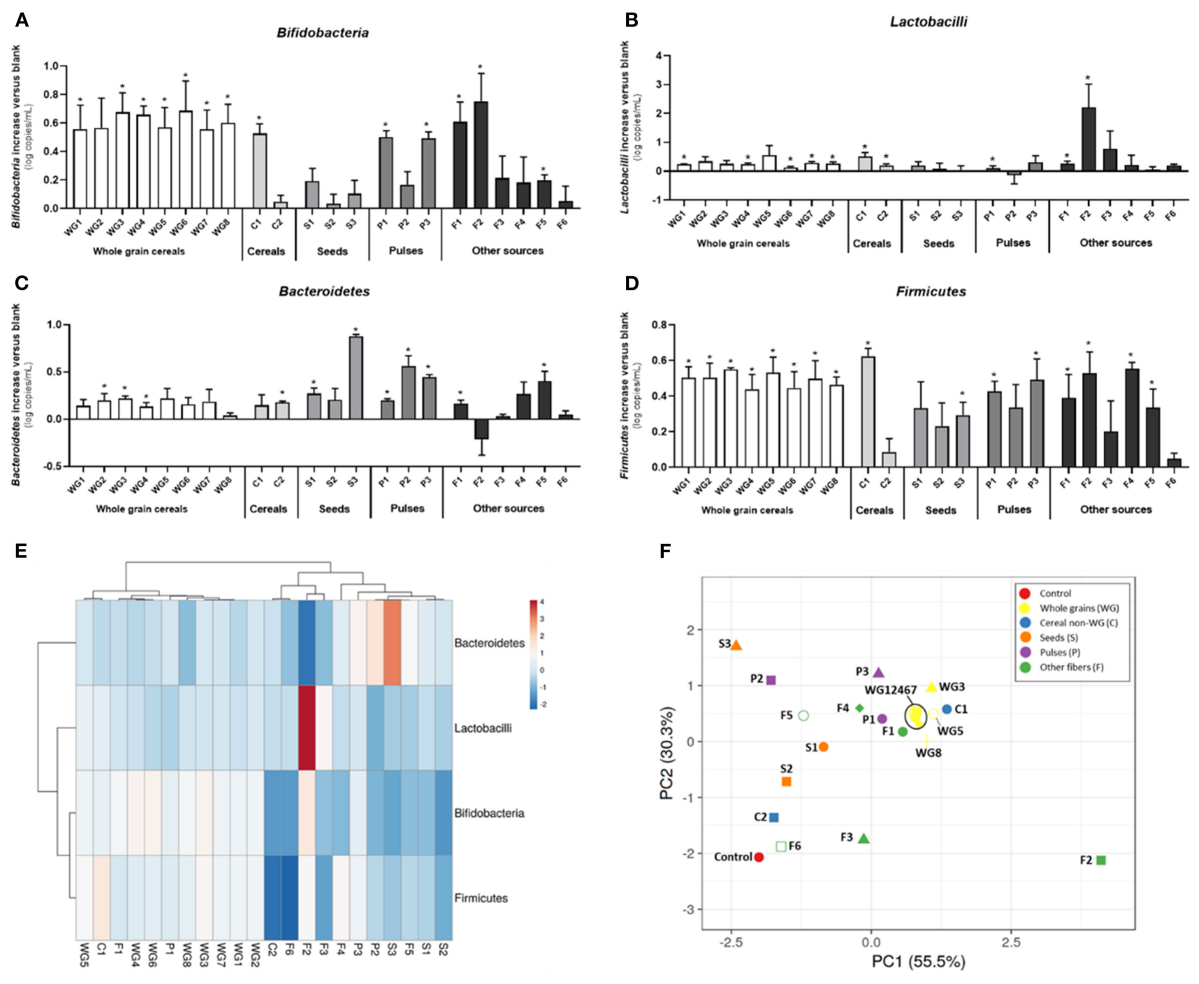
Effect of dietary fibers on absolute abundance of bifidobacteria, lactobacilli, Bacteroidetes, and Firmicutes. Bars represent the increase of bifidobacteria **(A)**, lactobacilli **(B)**, Bacteroidetes **(C)**, and Firmicutes **(D)** vs. the control condition (average of three donors, technical triplicate per donor ± SD) after exposure to different products for 48 h. Heatmap of Pearson's correlation analysis between absolute abundance of bifidobacteria, lactobacilli, Bacteroidetes, and Firmicutes and different fibers **(E)**. PCA of different bacterial groups after treatments **(F)**. WG1, whole grain millet; WG2, whole grain oat; WG3, medium grain whole brown rice; WG4, whole grain soft white wheat; WG5, whole grain barley; WG6, whole grain corn; WG7, waxy whole grain soft white wheat; WG8, waxy hulless barley; C1, oat beta glucan; C2, rice fiber; S1, whole brown flaxseed; S2, hemp seeds; S3, psyllium fiber; P1, whole brown lentils; P2, soy fiber; P3, pea fiber; F1, kiwi fiber; F2, inulin; F3, bamboo fiber; F4, konjac flour; F5, apple fiber; F6, algal beta glucan isolate.

Overall, fiber supplementation promoted bifidobacteria (*p* = 0.024) and Firmicutes (*p* = 0.001), with specific product differences. The whole grain group had a stimulatory effect on all four groups of bacteria, especially on bifidobacteria and Firmicutes (Table 2). WG1 (whole grain millet), WG4 (whole grain soft white wheat), WG6 (whole grain corn), WG7 (waxy whole grain soft white wheat), and WG8 (waxy hulless barley) significantly increased lactobacilli populations, while Bacteroidetes was stimulated by WG2 (whole grain oat), WG3 (medium grain whole brown rice), and WG4 (non-waxy whole grain soft white wheat) (Figure 3). These changes are observed in the PCA plot (Figure 3F) where whole grain products clustered together and were distant from the control condition.

**Table 2:** Effect of different groups of dietary fibers on specific members of gut microbiota.

	**Whole grain cereals**	**Cereals (non-whole grain)**	**Seeds**	**Pulses**	**Other fibers**
**Copies/ml (log units)**					
Bifidobacteria	**0.61** **±** **0.23**	0.29 ± 0.25	0.11 ± 0.14	**0.39** **±** **0.19**	0.33 ± 0.34
Lactobacilli	**0.29** **±** **0.23**	**0.35** **±** **0.25**	0.11 ± 0.24	0.09 ± 0.39	0.62 ± 1.00
Firmicutes	**0.49** **±** **0.12**	0.35 ± 0.29	**0.28** **±** **0.19**	**0.42** **±** **0.17**	**0.34** **±** **0.25**
Bacteroidetes	**0.16** **±** **0.12**	0.17 ± 0.12	0.45 ± 0.34	**0.40** **±** **0.19**	0.12 ± 0.25
Ratio F/B	**1.01** **±** **0.02**	1.00 ± 0.33	0.96 ± 0.03	0.98 ± 0.02	1.00 ± 0.03

*Values indicate the average ± SD of copies/ml in log units (n = 3 biological replicates and three technical replicates) obtained by specific qPCR for bifidobacteria, lactobacilli, Firmicutes, Bacteroidetes, and Firmicutes/Bacteroidetes ratio, in rΔ48 h. Statistically significant differences (p < 0.05) between different treatments and control condition are marked in boldface*.

Within the non-whole grain cereal group, only C1 (oat beta glucan) increased the levels of bifidobacteria, lactobacilli, and Firmicutes and clustered close to whole grains (Figures 3A,B,D,F). Contrarily, C2 (rice fiber) tended to increase the levels of Bacteroidetes and was closer to the control (Figures 3C,F).

When analyzed together, seeds did not affect the levels of bifidobacteria, lactobacilli, or Bacteroidetes (Figures 3A–C), but significantly promoted Firmicutes members (Figure 3D). S3 (psyllium fiber) exerted marked effects on Bacteroidetes, differing from the other seeds, which are both high-fat-containing products, with levels above 10 and 40%, respectively (Figures 3C,F).

Within the pulse group, P1 (whole brown lentils) and P3 (pea fiber) induced significant increases in bifidobacteria, lactobacilli, Bacteroidetes, and Firmicutes, clustering closer between them than to P2 (soy fiber), which only influenced Bacteroidetes group (Figures 3A–F).

Within the other fiber group, there was a larger intergroup variation. F1 (kiwi fiber) and F2 (inulin) increased bifidobacteria, lactobacilli, and Firmicutes. F1 exerted marked bifidogenic effects and clustered close to the whole grain products. Remarkably, F2 is differentiated from all the other samples in the PCA plot, likely due to the highest effect on lactobacilli populations. F3 (bamboo fiber) and F6 (algal beta glucan isolate) had milder effects on microbial composition and grouped closer to the control condition than other fibers. F4 (konjac flour) and F5 (apple fiber) clustered in between due to intermediate effects, with F5 stimulating bifidobacteria, Firmicutes, and Bacteroidetes and F4 only increasing Bacteroidetes and Firmicutes members. F6 did not affect any of the groups, and it is the closest sample to the control (Figures 3A–F).

WG3 (medium grain whole brown rice), WG8 (waxy hulless barley), P1 (whole brown lentils), and F2 (inulin) significantly increased the Firmicutes/Bacteroidetes ratio compared to control (Supplementary Figure 1). On the contrary, S3 (psyllium fiber) significantly decreased the ratio due to a strong effect on Bacteroidetes group. In summary, a ranked effect on bifidobacteria was observed for WG>P≈S≈O, while Firmicutes was modified by WG≈P>C≈O>S. Lactobacilli showed a different trend, with O>C>WG>S>P, while Bacteroidetes changes ranged as follows: S>P>C>WG>O.

Supplementary Table 5 shows the effect of different fibers on different bacterial members' modulation for the three different donors individually.

### A Large Variety of Dietary Fibers Are Highly Fermentable and Induce Short-Chain Fatty Acid Production by Gut Microbiota of Healthy Donors *in vitro*

When considering the different products in fiber groups, whole grains and pulses induced the highest pH drop and gas production, with significant changes of pH (−0.5 ± 0.1, *p* < 0.001, and −0.5 ± 0.2, *p* = 0.03, respectively) compared to control (−0.2 ± 0.01) (Table 3). Gas pressure vs. control reached levels of 50.6 ± 6.9 kPa for whole grains (*p* < 0.001) and 51.3 ± 7.8 kPa for P (*p* = 0.001).

**Table 3:** Effect of different groups of dietary fibers on bacterial metabolic markers.

	**Control**	**Whole grain Cereals**	**Cereals (non-whole grain)**	**Seeds**	**Pulses**	**Fibers**
pH	−0.2, 0.0	**−0.5** **±** **0.1**	−0.4, 0.2	−0.3, 0.2	–**0.5** **±** **0.2**	−0.5, 0.3
Gas (kPa)	28.2, 1.1	**50.6** **±** **6.9**	42.3, 13.4	**42.1** **±** **8.4**	**51.3** **±** **7.8**	**44.7** **±** **12.7**
Lactate (mM)	1.2, 0.1	**8.3** **±** **3.6**	5.5, 5.6	1.8, 0.7	3.5, 2.4	4.5, 6.9
Acetate (mM)	22.4, 4.2	**45.0** **±** **4.0**	33.9, 10.8	**32.8** **±** **6.4**	**41.2** **±** **9.7**	**34.6** **±** **8.8**
Propionate (mM)	8.0, 2.1	16.0, 3.6	13.7, 6.3	**16.4** **±** **4.1**	**17.3** **±** **7.1**	12.2, 3.8
Butyrate (mM)	3.1, 2.1	7.3, 4.6	6.0, 5.7	4.3, 1.7	5.6, 3.3	4.5, 2.4
Branched FA (mM)	1.9, 1.2	1.6, 1.4	1.8, 1.3	2.2, 1.6	1.7, 1.3	1.6, 1.2
Total SCFA (mM)	36.5, 9.1	70.6, 5.8	56.2, 17.9	56.3, 11.8	66.4, 17.8	53.5, 13.1

*Values indicate the average ± SD of different markers (n = 3 biological replicates and 3 technical replicates, rΔ48 h). Statistically significant differences (p <0.05) between different treatments and control condition are marked in boldface*.

The highest gas production and pH decrease were observed for F2 (inulin), while the lowest effect on pH and gas production was observed for S2 (hemp, −0.16 ± 0.01) and C2 (rice fiber, 31.4 ± 3.9 kPa), respectively.

In general, fibers induced a significant increase (ANOVA, *p* = 0.03) in lactate, with an average value of 5.5 ± 5.1 mM. When considering fiber groups, the highest net acetate levels were observed for the group of whole grain products (8.25 ± 3.58 mM, *p* = 0.045) compared to control (1.24 ± 0.14 mM), however with a high intragroup variability.

**Figure 4:**
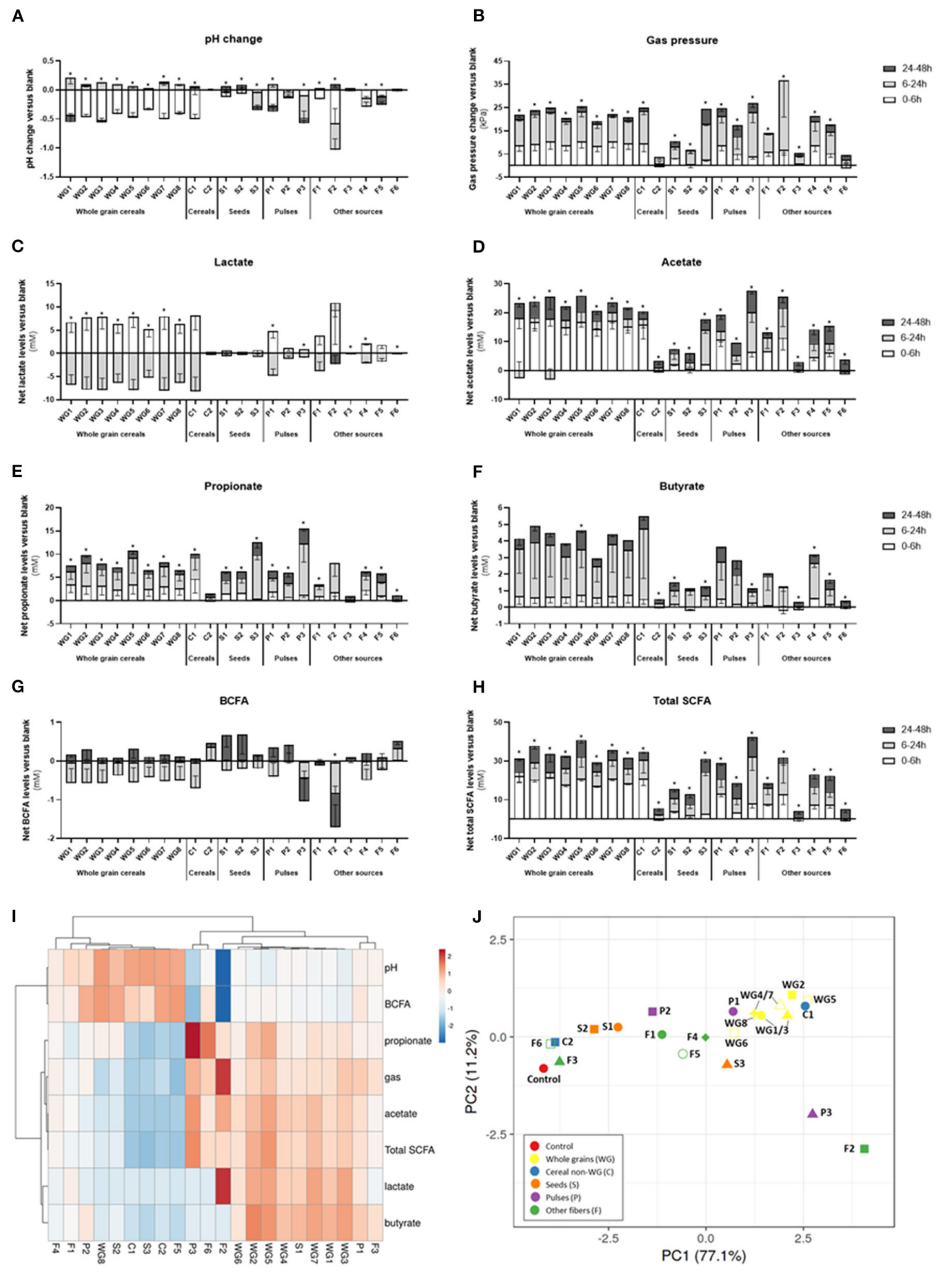
Effect of dietary fibers on microbial activity. Bars represent the net values of pH **(A)**, gas pressure **(B)**, lactate **(C)**, acetate **(D)**, propionate **(E)**, butyrate **(F)**, branched-chain fatty acids **(G)**, and total short-chain fatty acids **(H)**, normalized vs. control levels (average ± SD *n* = 3 donors; three technical replicates for each donor) after exposure *in vitro* of fecal samples from three healthy individuals to dietary fibers. Stacked bars indicate the metabolite production at different time points. Heat map of Pearson's correlation between different metabolic markers and treatments **(I)**. PCA of metabolic markers **(J)**. WG1, whole grain millet; WG2, whole grain oat; WG3, medium grain whole brown rice; WG4, whole grain soft white wheat; WG5, whole grain barley; WG6, whole grain corn; WG7, waxy whole grain soft white wheat; WG8, waxy hulless barley; C1, oat beta glucan; C2, rice fiber; S1, whole brown flaxseed; S2, hemp seeds; S3, psyllium fiber; P1, whole brown lentils; P2, soy fiber; P3, pea fiber; F1, kiwi fiber; F2, inulin; F3, bamboo fiber; F4, konjac flour; F5, apple fiber; F6, algal beta glucan isolate.

Total SCFA were consistently increased in all the groups (53.5–70.6 mM, ANOVA *p* < 0.0001) when compared to the control condition (36.5 ± 9.1 mM). The highest values of SCFA were observed for WG>P>C≈S>F (Figure 4). Concretely, acetate was significantly increased in all fiber groups, reaching levels from 32.8 ± 6.4 mM in seeds to 45.0 ± 4.0 mM in whole grains (Table 3). The highest and lowest levels of acetate were consistently observed for F2 (inulin, 47.9 ± 4.3 mM) and F3 (bamboo fiber, 24.6 ± 4.4 mM), respectively (Figure 4). Fibers had a similar trend in increasing propionate levels (ANOVA, 12.2 ± 3.8 mM, *p* < 0.001; Table 3) compared to control (8 ± 2.1 mM); however, it was only significant for seeds (16.4 ± 4.1 mM, *p* = 0.007; Table 3). The highest increase in propionate was induced by P3 (pea fiber, 23.5 ± 9.5 mM), while the lowest effect on propionate production was observed for F3 (bamboo fiber, 8.6 ± 1.4 mM) (Figure 4).

Regarding butyrate, it was a trend to increase levels by the supplementation of fibers (5.8 ± 3.7 mM) compared to control (3.1 ± 2.1 mM); however, the changes were not always significant due to a high interindividual variability on butyrate production at basal levels. In fact, the microbiota from one of the donors included in the study had low butyrogenic potential, with levels of butyrate of 1.9 ± 0.8 mM (average for all the treatments), compared with the other donors (5.9–8.8 mM) (Supplementary Table 6). When excluding this donor from the analysis, the group of whole grains induced an increase in butyrate (6.4 ± 1.1 mM) vs. the control condition (2.6 ± 0.7 mM), and concretely WG1 (whole grain millet, 6.6 ± 0.9 mM, *p* = 0.04), WG2 (whole grain oat, 7.3 ± 1 mM, *p* = 0.03), and WG3 (medium grain whole brown rice, 6.8 ± 0.1 mM, *p* = 0.04) showed a significant butyrogenic potential. Within the non-whole grain cereal group, C1 (oat beta glucan, 8.1 ± 0.6 mM, *p* = 0.014) also induced butyrate production, while the other groups or individual products did not significantly affect butyrate levels. Branched fatty acids were not significantly affected by the different fibers or groups of fibers, with the only exception of F2 (inulin) that reduced the BCFA levels (0.2 ± 0.2 mM) compared to the control (1.9 ± 0.2 mM).

## Discussion

This study assessed the effect of 22 commercially available food sources of fiber on gut microbial composition and function of three healthy human donors *in vitro*. Of 22 products, 19 were quickly fermented and induced changes in the gut microbiota, increasing health-promoting SCFA production and affecting key groups such as bifidobacteria and lactobacilli, while three products were more resistant to bacterial degradation and had minor effects on gut microbiota.

We tested a wide variety of fiber sources, including whole grain cereals, cereals (non-whole grain), seeds, pulses, and other fibers; however, a high heterogenicity in terms of composition was observed within each group. The groups with nutrients available for absorption in the small intestine were predigested, with intestinal absorption mimicked by a dialysis approach, to ensure that non-digestible compounds, and not the source of easily available sugars derived from product digestion, were the driver of microbial shifts in the colonic fermentation.

The group of whole grain products had a consistent effect in increasing acetate, lactate, and propionate after short-term incubations, together with the stimulation of bifidobacteria and lactobacilli. Firmicutes members were fueled by whole grain products, while Bacteroidetes group was, for most of the whole grain fibers, not significantly affected. These results are in agreement with human intervention studies showing that whole grains can impact fecal microbe levels; e.g., wheat, oat, and corn can increase *Bifidobacterium* levels, and whole grain wheat and oat can also increase *Lactobacillus in vivo* (32, 33). Whole grain interventions are inconsistent in their effects on SCFA concentrations in feces, with some showing slight or no effects (34, 35). The reason for this discrepancy is that most SCFAs are absorbed by the colonic epithelium or cross-feed colonic bacteria, resulting in only 5–10% of SCFAs being excreted in feces. In that sense, portal SCFAs have been reported, and multiple dietary fiber interventions in human and animal studies, including arabinoxylan, whole grain rye, or resistant starch diets among others, have shown a significant impact of dietary fiber on portal levels of SCFAs (36–38). Remarkably, most of the research on dietary fiber has been performed using common grain cereals such as wheat, barley, or rice, or purified products as arabinoxylans or inulin, while less information is available on the effect of waxy cereals, millet, or non-standard fibers sources on human gut microbiota. We observed a potential beneficial effect of most of the products used in this research on human gut microbiota, considering bifidobacteria, and lactobacilli boost and SCFAs and lactate production as specific prebiotic indicators.

Lactate was one of the metabolites specifically and consistently induced by whole grains, together with P1 (whole brown lentils) and F2 (inulin). Lactate is produced by many microbial members of the human gut, including lactic acid bacteria and bifidobacteria. lactate can influence colonic pH and has been shown to inhibit the growth of some pathogenic bacteria, including *Escherichia coli* (39). In addition, it serves as a substrate for lactate-utilizing bacteria, acting as a cross-feeding molecule in butyrate or propionate production by some members of the Firmicutes phylum (39). Therefore, an indirect benefit of increased lactate production by whole grains, likely caused by bifidobacteria and lactobacilli boost, is the production of health-promoting metabolites. Butyrate is the main source of energy for colonocytes, and an impairment in butyrate production may play a role in intestinal inflammation (40, 41). On the other hand, a positive correlation was found between Bacteroidetes members and propionate, a key metabolite in energy metabolic homeostasis and gut–liver crosstalk (42).

The effects on fermentation profiles and gut microbiota modulation of oat β-glucan were similar to those observed in whole grain cereals, despite the amount of dietary fiber in oat beta glucan being approximately five times higher. Oat β-glucans are classified as prebiotic soluble fibers with recognized health benefits (43, 44). β-Glucans are also present in wheat and barley, so it is hypothesized that some of the similar effects on gut microbiota may be mediated by this soluble fiber. Whole grain cereals can contain multiple types of fiber (such as glucofructans, hemicelluoses (pentosans), cellulose, and hemicellulose) and other potentially microbe-accessible substrates (like lignin, protein fractions, phenolic compounds, waxes, saponins, phytates, or phytosterols, among others) that can be intimately associated with plant polysaccharides (21), having a major impact on gut microbiota. Further characterization of insoluble and soluble fiber fractions in different products will shed light on the fractions having a major effect on gut microbiota ecology, supporting our theory that diversification of dietary sources of fiber might have a beneficial effect on host health through differential metabolic and compositional modulation of the gut microbiota.

The products with the lowest fermentability were rice fiber, bamboo fiber, and algal beta glucan isolate. These products were high in fiber, rich in either soluble (algal beta glucan) or insoluble fiber, with a limited amount (<1%) of other macronutrients such as protein, fat, sugars, or other non-fiber/sugar carbohydrates. This group seems to be undigested by the enzymatic repertoire in the human gut microbiota; however, a larger screening including more donors would be required for consistent conclusions. Although largely unfermented, this does not imply that they lack potential physiological benefit. Recently, bamboo shoot fiber has shown the ability to prevent obesity in high-fat-fed mice through the modulation of the gut microbiota (45). Fiber derived from the shoot or mature bamboo may differ in structure and composition; also, rodent microbiota and digestive processes are different from those in humans (46). With similar levels of fiber, psyllium had a markedly different effect on gut microbiota. Psyllium fiber had the highest effect promoting Bacteroidetes, potentially linked to the observed increase in propionate. Members of Bacteroidetes can produce propionate from hexoses through the succinate pathway (47); however, other members of the gut microbiota not analyzed in this study may be also related to propionate production after psyllium supplementation. Full characterization of microbial shifts through 16S rRNA sequencing, shotgun metagenomics, or metabolomic approaches is proposed for further studies.

The interindividual variability on taxonomic and functional composition of the gut microbiota is a key element in responses to dietary interventions (48). Indeed, human intervention studies using common prebiotic fibers do not present consistent results across individuals (48). Cantu-Jungles and Hamaker proposed a classification of dietary fibers based on their specificity to gut microbes, with two extremes defined as low-specificity fibers (e.g., inulin) easily available and fermented by many colonic microorganisms, and high-specificity fibers (e.g., insoluble glucans) only accessible and degradable by few bacteria. The authors proposed predictable shifts on gut microbiota when supplementing high-specific fibers, as only few members of the gut microbiota could benefit, while larger interindividual variability in low-specificity fiber intervention is expected (48). Here, we propose an approach including both specific and non-specific fibers derived from the plant origin, as a tool to promote beneficial members of the gut microbiota through nourishing the gut ecosystem with multiple dietary sources of fiber and carbohydrates. The effect of plant-based diets has been evaluated in an extensive study (11,336 human participants), concluding that the gut microbiota of individuals with a large intake of plant-based foods (> 30 different plant types per week) is more diverse than the gut microbiota of low-plant consumers (<10 different plant types per week) (49). Incorporating different sources of cereals from multiple origins could benefit both microbial diversity and specifically some key members of the human gut microbiota such as bifidobacteria and lactobacilli.

We analyzed three different microbial ecosystems derived from healthy donors in response to dietary fiber addition and obtained similar trends for all the donors. We identified the limitation of using a low number of donors and proposed larger screening with the most promising dietary fibers, including more donors to confirm our results. Further studies, including multiple fibers and human-derived disease-dysbiotic microbial communities, like occurring in obesity, diabetes, or inflammatory bowel disease, would provide more evidence on fiber diversity on microbial modulation of dysbiotic gut ecosystems.

Remarkably, bifidobacteria populations were stimulated in all the donors by all whole grains, brown lentils, pea fiber, kiwi, and apple fiber. The potential to promote specific individual bifidobacteria within individual gut ecosystems with diet-based solutions may benefit gut homeostasis “from the inside,” targeting residential strains already co-evolved with the host (50). Due to the relevance of bifidobacteria populations on human gut colonization and health, dietary interventions intended to diversify the sources of carbohydrates with bifidogenic potential may be beneficial for general consumers, but also for specific groups such as pregnant women, infants, and children, different disease states, and antibiotic-induced dysbiotic populations.

Previous research using mice models has shown that fiber deprivation triggers the colonic microbiota to utilize host-secreted glycoproteins as a nutrient source (51), inducing a loss of diversity of the mucus-associated microbiota and depleting the mucus layer (52). Alterations in the mucosal environment were linked to higher colonization of mucosal pathogens and disease development (52).

In addition to the relevant effect of fiber sources on mucosal health, complex fiber structures such as wheat bran can serve as a microenvironment to be colonized by specific degraders (53). The presence of multiple subecological niches within the gut would allow for niche specialization and diversification, while it is recognized that higher fiber promotes greater microbiome diversity (54). Enlarging the functional and structural composition of the gut microbiota is linked to the capacity of the ecosystem to recover from a perturbation, defined as resilience (55), keeping the gut ecosystem as a stable entity with potential influence on host homeostasis. Since we observed the ability of different food sources of fiber to modulate gut microbiota function and structure in different ways, it is proposed to promote the consumption of a variety of sources of fiber to nourish and maintain a diverse and resilient ecosystem, therefore promoting beneficial effects on host health.

The authors acknowledge significant limitations associated with this research, including (i) limited number of donors; (ii) short-term evaluation of fiber effect in static conditions; (iii) the microbiota analysis based on qPCR data of specific members of the gut microbiota offering less resolution than complete 16S rRNA gene sequencing; and (iv) lack of full characterization of nutritional parameters such as specific fiber structure or micronutrient contents.

Despite these limitations, our *in vitro* approach limited the interference of variables like diet on gut microbiota modulation, allowing for a direct assessment of fiber sources on different microbial parameters. We incorporated individual samples from three different donor models, combined with a large variety of dietary sources, some of them with limited published information available, increasing our knowledge about the fiber effect on gut microbiota and potentially on human health.

## Data Availability Statement

The original contributions presented in the study are included in the article/Supplementary Material, further inquiries can be directed to the corresponding authors.

## Ethics Statement

The studies involving human participants were reviewed and approved by University Hospital Ghent (reference number B670201836585). The patients/participants provided their written informed consent to participate in this study.

## Author Contributions

PV and AB were involved in conceptualization. MC, PV, JG, and AB were involved in data curation. PV, MC, and JG were involved in formal analysis. PV and AB were involved in funding acquisition. PV and JG were involved in methodology. PV and MM were involved in project administration. ER and AB were involved in utilizing resources. MM was involved in supervision. MC and PV were involved in visualization. MC was involved in writing the original draft. MC and AB were involved in writing the review and editing. All authors contributed to the article and approved the submitted version.

## Conflict of Interest

AB and ER are employees of the Kellogg Company. PV was employed by ProDigest. MC and JG are employed by ProDigest. MM is CEO of ProDigest. The authors declare that this study received funding from Kellogg Company. The funder had the following involvement in the study: conceptualization, funding acquisition, data curation, utilizing resources, writing and editing.
